# Systematic evaluation and statistical modeling of dual centrifugation and high-pressure homogenization as promising methods for scalable preparation of polymeric nanoparticles

**DOI:** 10.1186/s11671-026-04808-y

**Published:** 2026-07-17

**Authors:** Amr Ehab Kamel, Stefanie Klein, Lisa Schöne, Sara Gabr, Udo Bakowsky, Jens Schäfer

**Affiliations:** 1 Department of Pharmacy, Institute for Pharmaceutics and Biopharmaceutics, Philipps-Universität Marburg, Robert-Koch-Str. 4, 35037 Marburg, Germany; 2 Department of Pharmaceutics and Pharmaceutical Technology, Faculty of Pharmacy, Heliopolis University, Cairo, Egypt; 3 Department of Pharmacognosy, Faculty of Pharmacy, Heliopolis University, Cairo, Egypt

**Keywords:** Nanobiomaterials, Design of experiments, Scalable manufacturing, Precision medicine, Process optimization, Response surface methodology

## Abstract

Nanobiomaterials have emerged as critical platforms for modern therapeutics, offering targeted delivery, controlled release, and improved performance. Among these, polymeric PLGA nanoparticles (PLGA-NPs) are widely studied due to their tailorability, biocompatibility, biodegradability, and regulatory acceptance. However, their clinical translation depends on robust manufacturing approaches that support scalability, reproducibility, and continuous manufacturing. Conventional methods, such as high-shear homogenization, suffer from limitations, including high energy input, process sensitivity, and contamination risk. This study presents a comprehensive experimental and statistical approach to systematically compare three PLGA-NPs preparation methods: high-shear homogenization (HSH), dual centrifugation (DC), and high-pressure homogenization (HPH). A full factorial Design of Experiments (DoE) combined with Response Surface Methodology (RSM) evaluated the effects of formulation and process parameters, including PVA and PLGA concentrations, homogenization speed, bead diameter, and pressure on Critical Quality Attributes (CQAs; particle size, polydispersity index, and zeta potential). HSH produced uniform nanoparticles with narrow size distribution but was highly process-sensitive. DC showed more variability in results, while HPH achieved consistent results. PVA concentration was the dominant factor across all methods. In conclusion, DC and HPH emerged as viable candidates for scalable nanoparticles manufacturing.

**Graphical abstract:**

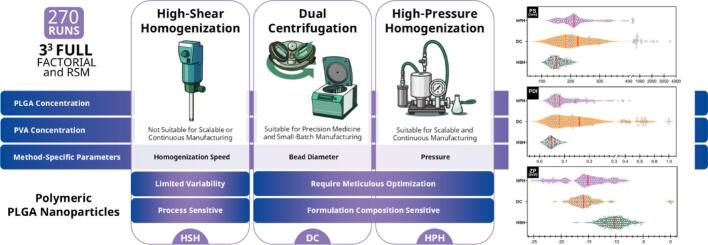

**Supplementary Information:**

The online version contains supplementary material available at 10.1186/s11671-026-04808-y.

## Introduction

Nanobiomaterials have emerged as critical platforms for modern therapeutics, enabling enhanced drug delivery with unprecedented precision. Their nanoscale dimensions provide unique physicochemical properties that enable modified drug release, significant targeting capabilities, reduced systemic toxicity, improved distribution, and the overcoming of systemic barriers [1, 2]. These advantages have positioned biomaterials-based nanoparticles (NPs) as a cornerstone of nanomedicine, with applications ranging from gene therapy and hormone delivery to next-gen vaccines, theranostics, and biomarker-based disease management [3–6]. Among the various types of nanobiomaterials, polymeric nanoparticles have drawn significant attention due to their biodegradability, tunable surface chemistry, and ability to encapsulate both hydrophilic and hydrophobic drugs [2, 4]. Poly(lactic-co-glycolic acid) (PLGA) stands out as the most widely used polymer in NPs formulations owing to its European Medicines Agency (EMA) and Food and Drug Administration (FDA) approval, biocompatibility, and well-characterized degradation profile [6, 7]. PLGA undergoes hydrolysis into lactic acid and glycolic acid, which are metabolized via the citric acid cycle (Krebs cycle), minimizing toxicity [7]. These properties make PLGA-based nanoparticles an appealing platform for the research and development of sustained drug delivery systems and nanomedicine [4, 6].

The manufacturing process of PLGA nanoparticles (PLGA-NPs) remains a serious hurdle for their clinical translation due to the intricate interactions between formulation and process parameters that often lead to high batch-to-batch variability [8, 9]. Even slight variations in Critical Process Parameters (CPPs), like polymer and stabilizer concentrations, energy input, mixing time, or equipment configuration, can translate into substantial change in physicochemical characteristics affecting the therapeutic performance of PLGA-NPs [8]. This necessitates tighter control to ensure consistently meeting the Quality Target Product Profile (QTPP). Regulatory bodies, like the International Conference on Harmonization (ICH), FDA, and EMA, are enforcing a wide-scale adoption of Quality-by-Design (QbD) [8, 10–13]. Through QbD implementation, Critical Process and Material Parameters (CPPs and CMPs) can be systematically optimized to achieve the desired Critical Quality Attributes (CQAs), such as application-specific particle size, monodispersity, surface charge, and drug loading [2, 8, 14]. In turn, this contributes to facilitating reproducibility, scalability, continuous manufacturing, and streamline production.

Various techniques can be used to prepare PLGA-NPs, including, but not limited to, solvent evaporation, emulsification-solvent evaporation, solvent displacement, salting-out, nanoprecipitation, and microfluidics [2, 6, 9, 15, 16]. At the production level, these techniques are translated into distinct preparation and processing methods, such as ultrasonication, high-shear homogenization, or high-pressure homogenization [9, 17]. Among these methods, high-shear homogenization (HSH) remains widely used for PLGA-NPs preparation and is often regarded as the conventional gold standard [14]. HSH involves emulsification followed by solvent removal to achieve nanoparticle formation [3]. However, this method is often associated with batch processing and potential contamination risks arising from the prolonged contact between formulations and metal probes under high shear forces, causing serious challenges from scalable and continuous manufacturing standpoint [14]. To address the limitations of batch processing, there is also increasing emphasis from regulatory bodies on adopting continuous manufacturing [14, 18, 19].

Recent advances in continuous manufacturing address two fronts: high-throughput scale-up production and scale-down for precision medicine. For scale-up manufacturing, high-pressure homogenization (HPH) offers a continuous approach to large-scale production of various nanoparticles, providing consistent particle size and morphology while maintaining high throughput and energy efficiency [20, 21]. HPH employs pressure to induce shockwaves and intense mechanical forces resulting in cavitation that leads to size reduction in a more controlled and continuous manner [22]. For small-scale manufacturing, dual centrifugation (DC) offers a promising batch-based solution, avoiding complications of novel methods such as microfluidics [23–25]. DC employs controlled centrifugal forces by rotating a sample vial in the presence of inert beads along two distinct axes, generating intense and frequent particle movements [23]. These unique attributes facilitate in-vial homogenization and nanomilling, allowing fine control over nanoparticle formation with precise size distributions. However, DC relies on in-vial homogenization, therefore it is not suited for continuous manufacturing [23]. Nonetheless, it can be an ideal candidate for personalized medicine applications where sterile, patient-specific formulations are required.

While DC has been used to prepare lipid-based nanoparticles and nanocrystals, its applications in polymeric NPs remain greatly unexplored with a notable lack of literature on the topic to date [23]. Therefore, this study utilizes a QbD approach to perform the first systematic comparison between DC and two established PLGA-NPs preparation methods (HSH and HPH). For HSH, it has been shown that, in addition to the PVA and PLGA concentrations, the homogenization speed also has a significant influence on particle size [16]. Analogously to this process parameter in HSH, bead size was used as an important process parameter for DC and pressure for HPH. These methods employ a top-down technique, where mechanical forces break down polymeric emulsions into nanoemulsions, followed by removing organic solvents and collecting nanoparticles [6, 14, 23]. The basis for the comparison is the ability of the methods to meet a predefined set of CQAs in terms of particle size, monodispersity and zeta potential, then by identifying the optimal formulation and process parameters for each method within the design space, the study seeks to establish a reliable and scalable framework for PLGA-NPs preparation using DC and HPH as viable and regulatory-compliant alternatives for HSH.

## Materials and methods

### Materials

Poly(lactic-co-glycolic acid) (PLGA, acid-terminated, 50:50 lactide-to-glycolide ratio, 0.45 dL/g, PURASORB^®^ PDLG 5004 A) was a gift from Corbion (Gorinchem, Netherlands). Polyvinyl alcohol (PVA, 87–89%, MW 31,000–50,000 g/mol, KURARAY POVAL^®^ 4–88) was a gift from Kuraray Co., Ltd. (Tokyo, Japan). Ethyl acetate (purity ≥ 99.5%) was obtained from Sigma-Aldrich (Merck KGaA, Darmstadt, Germany). Ultrapure water was freshly acquired using a PURELAB flex system (ELGA Veolia, High Wycombe, UK).

### Design of experiment (DoE)

A 3^3^ full-factorial experimental design combined with response surface methodology (RSM) was employed to systematically assess the effects of key process and formulation parameters in HSH, DC, and HPH methods on PLGA-NPs characteristics. The design was augmented with sufficient replicate and center points to detect nonlinear interactions between factors, enabling reliable estimation of pure error and improved interpretability of formulation-process interactions.

The design for each preparation method included three numeric factors, each with three discrete levels, ensuring comprehensive exploration of the experimental space while maintaining statistical efficiency, as summarized in Table 1. The experimental levels were determined based on previous work form our laboratory and existing knowledge to ensure the quality of the resulting nanoparticles with minimal optimization steps [3, 7, 8, 16, 23, 26–28]. The first two factors (PVA and PLGA concentration) were formulation-specific, and their levels were consistent in all methods. The third factor was a method-specific Critical Process Parameter (CPP). For the HSH method, it was set to the homogenization speed (rpm). For the DC method, it was the bead diameter (mm), while for HPH, it was the pressure (psi).

**Table 1:** Breakdown of the experimental design factors and their levels

Factor	Name	Level [1]	Level [2]	Level [3]	Unit
A	PVA Concentration	1	3	5	% w/w
B	PLGA Concentration	4.0	14.5	25.0	mg/mL
C	Method-Specific				
	Homogenization Speed	12,000	15,000	18,000	rpm
	Bead Diameter	0.8	1.1	1.5	mm
	Pressure	10,000	15,000	20,000	psi

**Fig. 1:**
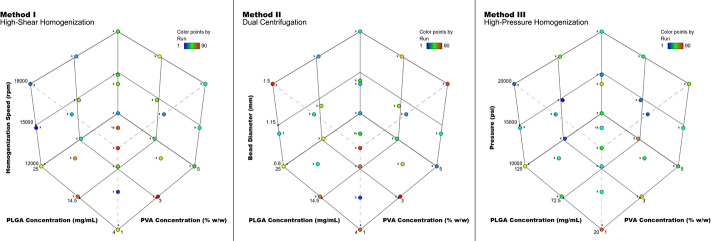
Distribution of randomized experimental points across the 3D design space. X-axes illustrate PVA concentration (1–5% w/w), Y-axes illustrate PLGA concentration (4–25 mg/mL), and Z-axes illustrate the different method-specific parameters (Factor C) from high-shear homogenization (HSH), dual centrifugation (DC), and high-pressure homogenization (HPH). The figure highlights the number of replicates per design point and center points, where each unique configuration was run in triplicates, except for the center point, which was repeated 12 times

The factorial design yielded 27 unique experimental configurations (Table 2) spanning the three numeric factors across their predefined levels. To enhance statistical robustness and improve the models’ reliability, the design was augmented by increasing the number of replicates, resulting in a total of 90 experimental runs. Each unique configuration was run in triplicate, except for the center point, which was repeated 12 times, resulting in 90 independently prepared samples (Table 2; Fig. 1). Samples were prepared according to the randomized run order generated by the design model to minimize systematic bias and account for uncontrolled sources of variability. The prepared PLGA-NPs samples were tightly sealed in labeled falcon tubes and stored in the laboratory refrigerator until physicochemical characterization tests were conducted.

**Table 2:** Breakdown of the 27 unique factorial configurations and their replicates spanning the different levels of experimental factors: PVA concentration (Factor A; % w/w), PLGA concentration (Factor B; mg/mL), and the method-specific parameter (Factor C). Factor C was the homogenization speed (rpm) in high-shear homogenization (HSH), bead diameter (mm) in dual centrifugation (DC), and pressure (psi) in high-pressure homogenization (HPH)

Config.	Replicates	Factor A	Factor B	Factor C	Factor C	Factor C
				HSH	DC	HPH
1	3x	1	4.0	12,000	0.8	10,000
2	3x	1	4.0	15,000	1.1	15,000
3	3x	1	4.0	18,000	1.5	20,000
4	3x	1	14.5	12,000	0.8	10,000
5	3x	1	14.5	15,000	1.1	15,000
6	3x	1	14.5	18,000	1.5	20,000
7	3x	1	25.0	12,000	0.8	10,000
8	3x	1	25.0	15,000	1.1	15,000
9	3x	1	25.0	18,000	1.5	20,000
10	3x	3	4.0	12,000	0.8	10,000
11	3x	3	4.0	15,000	1.1	15,000
12	3x	3	4.0	18,000	1.5	20,000
13	3x	3	14.5	12,000	0.8	10,000
14	12x	3	14.5	15,000	1.1	15,000
15	3x	3	14.5	18,000	1.5	20,000
16	3x	3	25.0	12,000	0.8	10,000
17	3x	3	25.0	15,000	1.1	15,000
18	3x	3	25.0	18,000	1.5	20,000
19	3x	5	4.0	12,000	0.8	10,000
20	3x	5	4.0	15,000	1.1	15,000
21	3x	5	4.0	18,000	1.5	20,000
22	3x	5	14.5	12,000	0.8	10,000
23	3x	5	14.5	15,000	1.1	15,000
24	3x	5	14.5	18,000	1.5	20,000
25	3x	5	25.0	12,000	0.8	10,000
26	3x	5	25.0	15,000	1.1	15,000
27	3x	5	25.0	18,000	1.5	20,000

### Preparation of PLGA-NPs

PLGA-NPs were prepared using three methods: high-shear homogenization, high-pressure homogenization, and dual centrifugation. These methods employ a top-down technique, where mechanical forces break down polymeric emulsions into nanoemulsions, followed by the removal of organic solvents and the collection of nanoparticles [6, 14, 23].

#### Preparation of aqueous and organic phases

Aqueous and organic phases were prepared for all methods according to the DoE. The aqueous phase was prepared by weighing and dissolving the specified amounts of PVA in ultrapure water to prepare 1%, 3%, and 5% w/w solutions. The solutions were filtered using 0.2 μm membrane filters and stored at 4 °C until use. The organic phase (PLGA solution) was freshly prepared by weighing and then dissolving the specified amounts of PLGA in 5 mL ethyl acetate in glass vials under continuous stirring to obtain PLGA solutions with concentrations of 4.0, 14.5, and 25.0 mg/mL.

#### High-shear homogenization (HSH)

PLGA-NPs were prepared following a modified emulsion–diffusion–evaporation method [3, 16, 26]. According to the DoE, 5 mL of the appropriate aqueous phase (PVA solution) was placed in a clean glass vial under continuous stirring. Then, 5 mL of the corresponding PLGA solution was slowly introduced using a syringe. The resulting o/w emulsion was immediately homogenized using Ultra-Turrax T25 digital equipped with SN-25-18G homogenization tool (IKA-Werke GmbH & Co. KG, Staufen, Germany) for 10 min at the speed designated for each sample by the DoE (12,000, 15,000, or 18,000 rpm). After homogenization, 40 mL of ultrapure water was added. The dispersions were stirred with 800 rpm on the IKA RT 10 power Multi-Stirring Plate (IKA-Werke GmbH & Co. KG, Staufen, Germany) at 20 °C overnight to evaporate the ethyl acetate, resulting in nanoprecipitation of PLGA-NPs. The evaporated solvent was replaced with ultrapure water the following day.

#### Dual centrifugation (DC)

The aqueous and organic phase solutions (PVA and PLGA solutions, respectively) were combined in a 1:1 ratio and mixed in a 2 mL screw cap DC vial (Sarstedt AG & Co. KG, Nümbrecht, Germany) according to the run order of the DoE. Yttrium-stabilized Zirconium oxide beads (Sigmund Lindner GmbH, Germany) were added in a 1:1 (w/w) ratio to vials based on the corresponding bead diameter in the DoE (0.8, 1.1, or 1.5 mm). The resulting pre-emulsion was then centrifuged using a ZentriMix 380 R centrifuge with a standard S-Rotor (Hettich GmbH & Co. KG, Tuttlingen, Germany) at 2350 rpm for 11 min at 4 °C to minimize degradation. After centrifugation, samples were transferred to new screw-cap vials and diluted by mixing with 2.4 mL of ultrapure water. They were then stirred on a multi-stirring plate with the same stirring speed as the HSH at room temperature until the ethyl acetate had evaporated entirely. Ultrapure water was added to each vial to compensate for solvent loss.

#### High-pressure homogenization (HPH)

Similar to the HSH method, the preparation method followed a modified emulsion-solvent-diffusion method. According to the DoE, equal volumes of both the aqueous and organic phase solutions (PVA and PLGA solutions, respectively) were mixed to form a pre-emulsion. The pre-emulsion was homogenized using the standard Avestin Emulsiflex C5 high-pressure homogenizer without custom modifications (Avestin Inc., Ottawa, Canada) at pressures specified by the DoE (10,000, 15,000, or 20,000 psi) over 5 complete cycles. Following homogenization, the emulsion was diluted with water and stirred with the same stirring speed as the HSH on the multi-stirring plate overnight to evaporate the remaining organic solvent [20].

### Physicochemical characterization

The particle size (PS), polydispersity index (PDI), and Zeta potential (ZP) of the prepared PLGA-NPs were characterized using Dynamic Light Scattering (DLS) and Laser Doppler Velocimetry (LDV) with a Zetasizer Nano ZS instrument (Malvern Panalytical, Kassel, Germany) in a disposable folded capillary cell (DTS1070, Malvern Instruments). For DLS analysis, the PLGA-NPs samples were diluted with ultrapure water at a 1:10 ratio and measured at a wavelength of 633 nm with a backscatter detection angle of 173°. The temperature was set to 25 °C, and the instrument was instructed to optimize the laser attenuation and measurement position automatically. Measurements were done in three runs, with each run comprising 10–100 subruns, each lasting 10 s. ZP was determined using LDV at a scattered light angle of 17°. The results (Z-average PS, PDI, and ZP) were calculated as the means of intensity distributions and reported with standard deviations.

### Statistical analysis and modeling

A comprehensive statistical analysis framework was established to ensure a thorough interpretation of the experimental data. The statistical approaches included factorial model validation, response surface methodology (RSM), and multi-parameter model optimization to identify optimal configurations. In this study, the p-value of < 0.05 was considered statistically significant, and a 95% confidence interval was applied for all analyses. Statistical analyses were executed using Stat-Ease 360 (Version 23; Stat-Ease, Inc., Minneapolis, MN, USA) and GraphPad Prism (Version 9; GraphPad Software, San Diego, California, USA).

#### Factorial model validation

The suitability and adequacy of the factorial and RSM models were evaluated using ANOVA, lack-of-fit tests, adjusted and predicted R^2^ values, and model adequacy (Adeq Precision), ensuring statistical validity in capturing the response trends [29–31]. The predicted R^2^ ($\:{\mathrm{R}}_{\mathrm{p}\mathrm{r}\mathrm{e}\mathrm{d}}^{2}$) provides an estimate of how well the model can be extrapolated to predict new data and reflects the model’s generalizability. The adjusted R^2^ ($\:{\mathrm{R}}_{\mathrm{a}\mathrm{d}\mathrm{j}}^{2}$) demonstrates the data fitting accounting for the number of model terms included. Unlike the predicted R^2^, the adjusted R^2^ can detect model overfitting. A difference of less than 0.2 between adjusted and predicted R^2^ was considered satisfactory for model reliability, exhibiting a good balance between fit and predictive capabilities [31]. Adeq Precision is a signal-to-noise ratio that indicates the model’s ability to navigate the design space. A value > 4 was required to ensure a sufficient signal for optimization [30–32].

#### Response surface methodology (RSM) analysis

RSM was employed to construct second-order polynomial models to analyze and interpret the interactions between experimental factors (CPPs; process and formulation parameters) and the responses (physicochemical properties: PS, PDI, and ZP) in each preparation method. The second-order models allow capturing both linear effects and potential curvature in the response trends, enabling a thorough understanding of how individual factors and their interaction could influence the critical quality attributes [31].

#### Identification of optimal configurations

Final RSM model optimization was performed through the desirability function in Stat-Ease 360 based on the RSM analysis to identify optimal configurations. Internal QTPP were defined to ensure obtaining physically stable and narrowly distributed samples with PS < 250 nm and PDI < 0.3 [33]. Accordingly, the optimal combination of formulation and process parameters was identified for each preparation method. Based on the QTPP, the process aimed to identify the best performing combinations of formulation and process parameters, meeting a set of strict equally-weighted CQAs, including a minimized PS within the range of 100–200 nm, a maintained monodispersity with a PDI between 0.01 and 0.1, and a strong negative ZP. A desirability score of > 0.6 was set to balance these parameters, ensuring optimal and reliable formulation selection. The desirability function combines multiple CQA criteria into a single composite score ranging from 0 (unacceptable) to 1 (ideal), based on the individual desirability of each response from the RSM-predicted values according to the defined goals and their weights [31, 32].

## Results

### Physicochemical Characterization

**Fig. 2:**
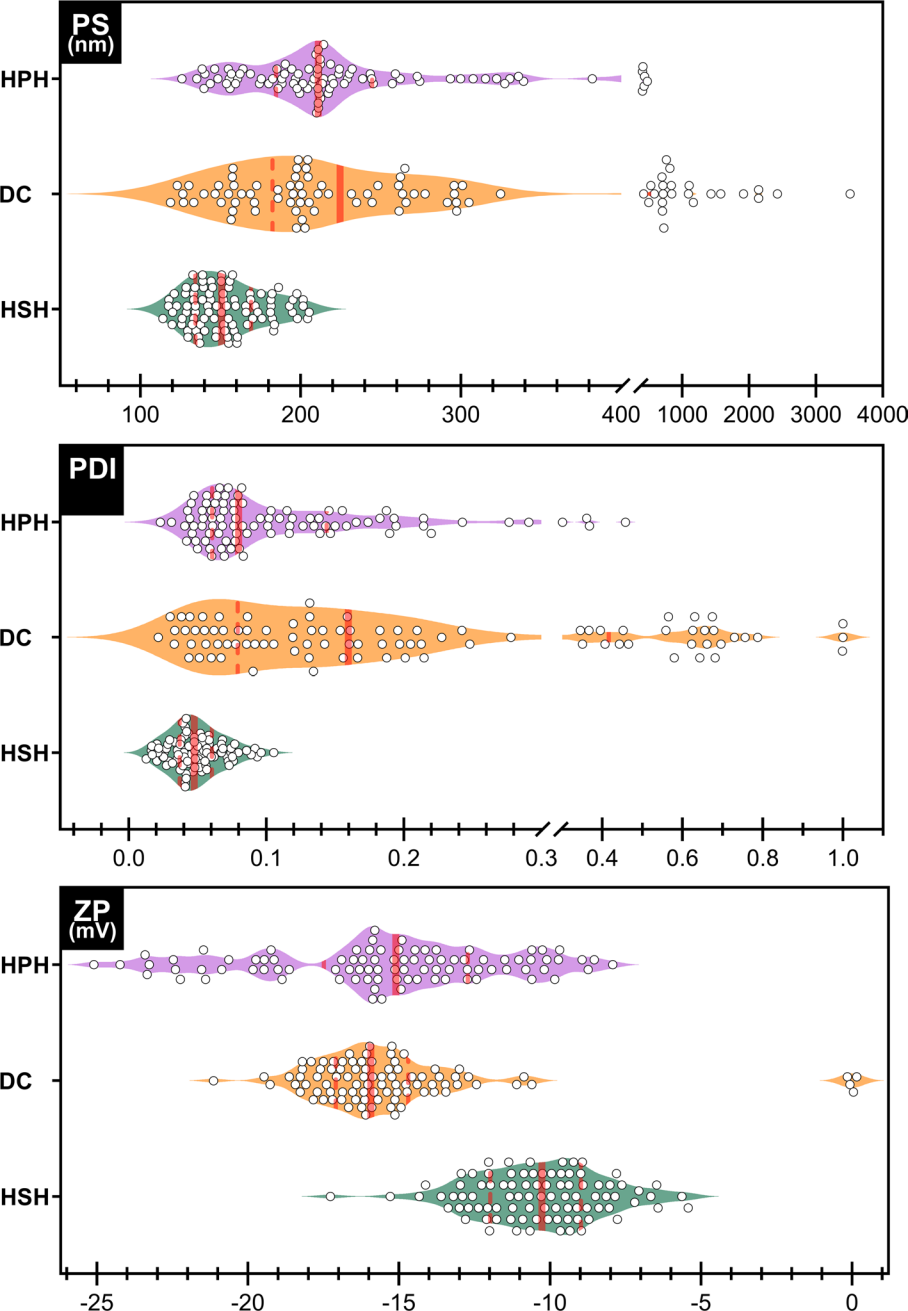
Violin plots showing the distribution of particle size (PS), polydispersity index (PDI), and zeta potential (ZP) for PLGA nanoparticles prepared using high-shear homogenization (HSH), dual centrifugation (DC), and high-pressure homogenization (HPH) across 90 experimental runs. Individual data points are staggered. The solid red line represents the median, while the dashed red lines indicate the first and third quartiles. The internal QTPP was defined at PS < 250 nm and PDI < 0.3

The systematic characterization of PLGA-NPs serves as the basis for understanding the relationship between process and formulation parameters (CPPs) and final CQAs. Particularly, PS and PDI are critical determinants of the quality and performance and therefore require systematic characterization and control [33]. Following the DoE, the characterization results were analyzed across all experimental runs to generate datasets for RSM analysis (Table S1, Fig. 2, and Fig. S1). Median and interquartile range (first and third quartiles) were selected as descriptors of central tendency and dispersion, as they are robust to the outliers inherent in an exploratory full-factorial design space and provide a statistically sound representation of method performance compared to mean-based metrics such as relative standard deviation, which are highly sensitive to extreme values [31].

Within these datasets, the particle size and polydispersity index results were the most informative. PLGA-NPs prepared by HSH demonstrated a high degree of particle size control and a narrow size distribution. Most samples clustered tightly around the median value (Fig. 2).

In contrast, PLGA-NPs prepared by DC showed greater variability in size and distribution. Nonetheless, the results remained within an acceptable range from the defined QTPP where samples with PS < 250 nm constituted 56% of the dataset, while those with PDI < 0.3 represented 70% of the dataset (Fig. 2). A subset of formulations exhibited larger particle sizes and broader PDI values, hinting at the presence of aggregates or insufficient size reduction. These observations reflect the need for further optimization or separation. The individual data points were flagged as potential outliers but were not excluded from analysis.

On the other hand, HPH exerted better control over CQAs and yielded PLGA-NPs with sizes and PDI profiles closer to HSH than DC. The median particle size distribution was relatively consistent and monodisperse, with the majority of formulations falling around the median value (Fig. 2). A small fraction of samples exhibited larger sizes, but the overall variability was limited and within the model’s tolerance.

Moreover, all the prepared PLGA-NPs in the three methods possessed a negative ZP, except for the outliers observed in the DC method, as illustrated in Fig. 2. The observed negative ZP can be attributed to the exposed carboxylic terminals of the PLGA polymer on the surface of nanoparticles that deprotonate in aqueous environment and impart a negative surface charge [34, 35]. Although the surface is practically coated by a layer of stabilizer molecules (PVA) that modulates the net charge, variations in surface coverage, adsorption density, or polymer-stabilizer interactions can influence the extent of negative charge observed across different samples [36].

Overall, the results indicated that HPH was the closest to HSH results in terms of median PS and PDI, with a broader variability in size but a narrower size distribution and optimal CQAs in comparison to the PLGA-NPs prepared by DC at the current levels for formulation and process parameters. These measurements were fed into the factorial DoE model for validation and statistical analysis to develop predictive models that correlate formulation parameters (PVA and PLGA concentrations) with method-specific parameters and predefined CQAs. No data points or suspected outliers were removed to maintain an unbiased analysis and complete comparative analysis. Moreover, the presence of extreme values at the boundaries of the design space was an expected outcome of a full-factorial DoE intentionally designed to explore the full parameter range, and their retention ensured robust model estimation and honest representation of method performance across the studied conditions [31, 32].

### Statistical analysis and modeling

#### Factorial model validation

The statistical analysis of the quadratic models for responses (PS, PDI, and ZP) across the three preparation methods revealed that the selected factors (CPPs) significantly influenced the responses (CQAs), as indicated by the highly significant model p-values (*P* < 0.0001). The quadratic RSM model was selected for all responses to enable response surface optimization, account for potential nonlinear relationships between factors and responses, and capture the main effects and possible interactions [31].

**Fig. 3:**
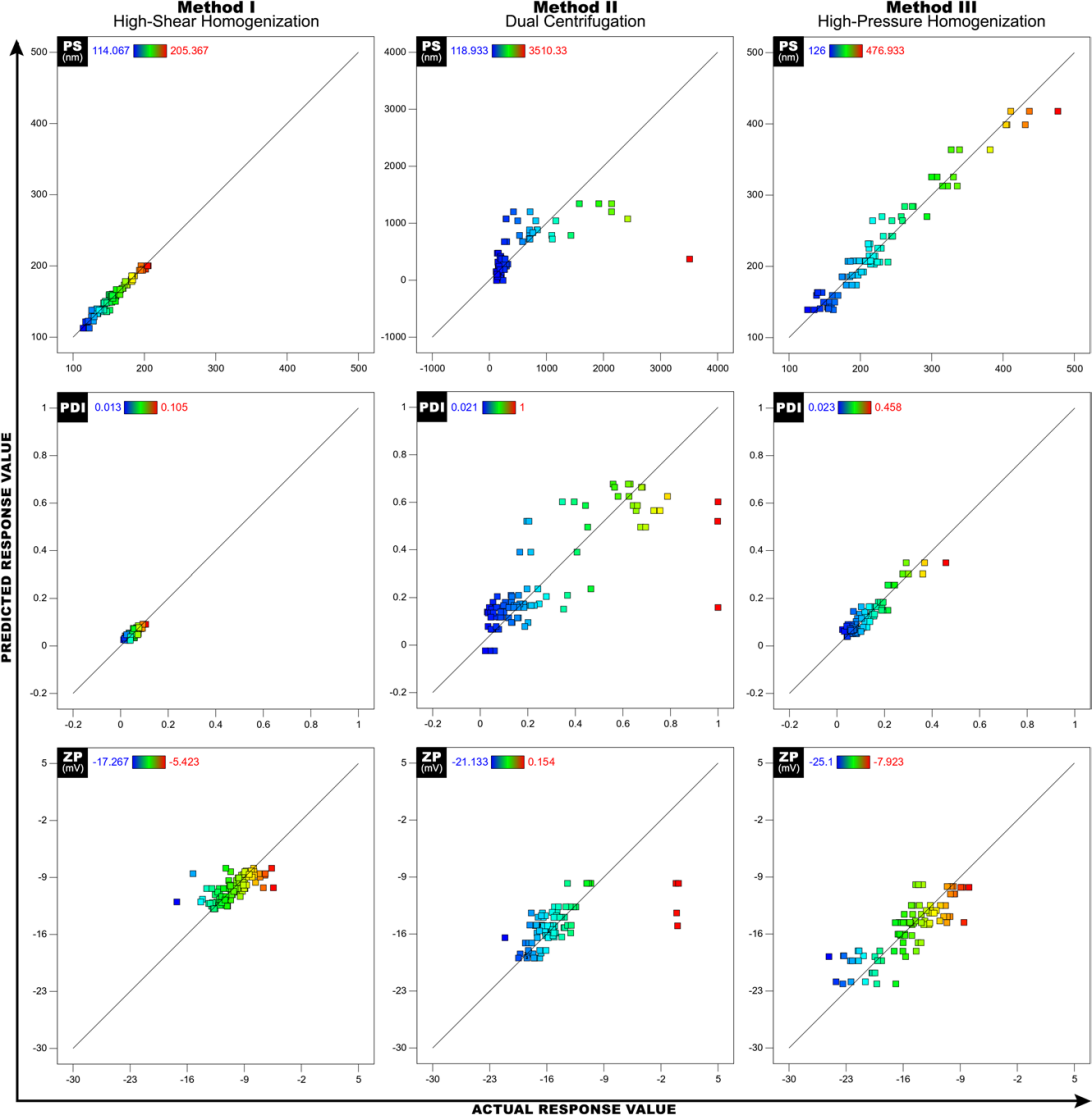
Predicted vs. Actual plots for particle size (PS), polydispersity index (PDI), and zeta potential (ZP) in high-shear homogenization (HSH), dual centrifugation (DC), and high-pressure homogenization (HPH) methods. The diagonal line represents the perfect agreement between predicted and actual values. HSH and HPH models showed high predictive accuracy with minimal deviations. DC models showed strong clustering apart from a few outliers (red points). Table 3 provides the adequacy and model fit statistics

In the high-shear (HSH) method, the predicted vs. actual plots (Fig. 3) confirmed that the RSM models exhibited high predictive accuracy, particularly for PS, with data points clustering tightly along the diagonal line (representing perfect agreement between predicted and actual values). This indicated a well-defined response to the change in formulation and process parameters. However, in ZP measurements, some variability was present at extreme values.

On the other hand, the DC models (Fig. 3) demonstrated more variability, as evident by the scattered data points along the diagonal line and the presence of extreme measurements and outliers, particularly for PS and ZP. Nonetheless, the model validation criteria (Table 3) emphasized the models’ predictive accuracy and suitability for exploring the design space. The observed deviations could be due to irregular nanoparticle formation, insufficient size reduction, or aggregation, which affect the quality of the nanoparticles and, consequently, the reliability of the models. However, no data points were excluded from the model analysis to maintain a fair, unbiased comparative ground between the three methods.

The high-pressure (HPH) models (Fig. 3) exhibited similar predictive accuracy to HSH models, indicating a strong correlation between actual and predicted measurements. However, the data points spanned longer ranges, especially for PS, suggesting the presence of further uncontrolled variables in the preparation method, which necessitates tighter optimization. The spread pattern was less prominent in PDI calculations, affirming the relatively narrow particle distribution.

Overall, this preliminary model validation highlighted the trending patterns in each preparation method and the extent to which they were optimized at the studied levels. The models were subjected to further statistical analyses to evaluate the influence of formulation and process parameters on each response variable and ensure their suitability for guiding formulation optimization.

#### Response surface methodology (RSM) analysis

**Fig. 4:**
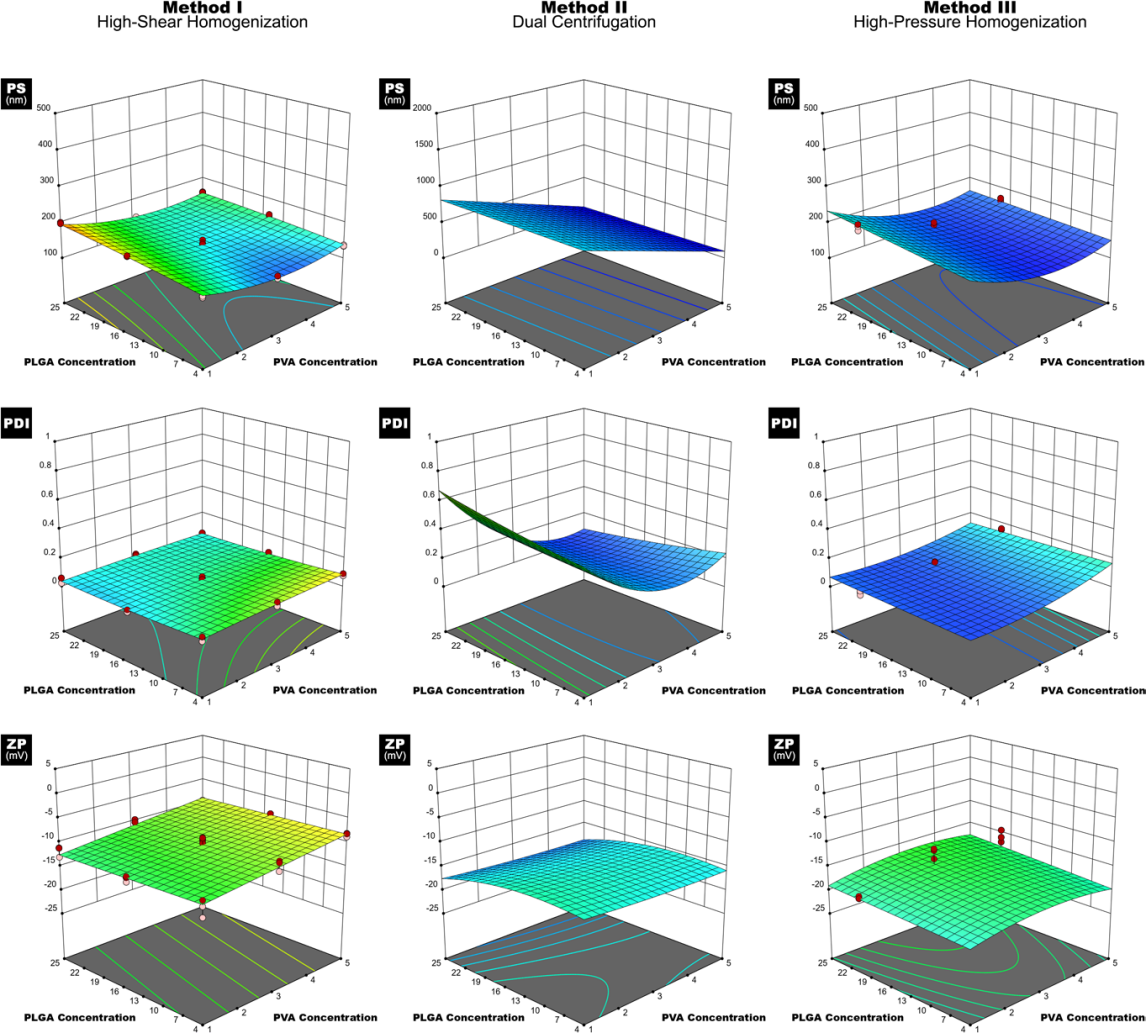
3D surface plots for the interaction between PVA and PLGA concentrations (Factors A and B, respectively) on the responses (PS, PDI, and ZP) in high-shear homogenization (HSH), dual centrifugation (DC), and high-pressure homogenization (HPH) methods. The level of the method-specific parameter (Factor C) was set to 15,000 rpm (Homogenization Speed in HSH),1.1 mm (Bead Diameter in DC), and 15,000 psi (Pressure in HPH) for visualization purposes. The significance and effects of Factor C are reported in Tables 4 and 5

The primary aim of the factorial experiments and response surface models was to identify the best performing combinations of formulation and process parameters and quantify their influence on PLGA-NPs characteristics within the studied design space. In this regard, the predictive accuracy beyond the explored space is outside the scope of this study. The analysis was intentionally restricted to experimentally evaluated conditions to preserve validity. Acknowledging this fact, the fitted RSM models effectively captured the interaction between formulation and process parameters (CPPs) and responses (CQAs) within the design space with sufficient statistical significance, supporting their use in optimization (Table 3; Fig. 4). The difference between $\:{\mathrm{R}}_{\mathrm{a}\mathrm{d}\mathrm{j}}^{2}$ and $\:{\mathrm{R}}_{\mathrm{p}\mathrm{r}\mathrm{e}\mathrm{d}}^{2}$ remained < 0.2 in all models. However, a significant lack-of-fit was observed in some models, as detailed later in their respective sections. Such lack-of-fit is commonly associated with nonlinear effects or higher-order interactions in complex formulation systems. This does not necessarily disqualify the models’ from being used in factor screening or identification of optimal configurations, especially when supported by other statistical tests [31]. Therefore, for the scope of this study, data interpretation, and response optimization, the models remain sufficiently reliable within the design space.

**Table 3:** Adequacy and fit statistics for RSM models of experimental responses: particle size (PS), polydispersity index (PDI), and zeta potential (ZP) in high-shear homogenization (HSH), dual centrifugation (DC), and high-pressure homogenization (HPH) methods

Method	$\:{\mathbf{R}}_{\mathbf{a}\mathbf{d}\mathbf{j}}^{2}$	$\:{\mathbf{R}}_{\mathbf{p}\mathbf{r}\mathbf{e}\mathbf{d}}^{2}$	Adeq precision	Model (*p*-value)	Model (F-value)	Lack of Fit (*p*-value)
*HSH*						
PS	0.952	0.946	51.001	****	195.0	**
PDI	0.651	0.597	17.215	****	19.45	ns
ZP	0.417	0.372	14.452	****	22.18	ns
*DC*						
PS	0.259	0.233	9.295	****	11.37	ns
PDI	0.608	0.567	13.002	****	16.35	ns
ZP	0.280	0.204	8.598	****	4.85	ns
*HPH*						
PS	0.942	0.931	46.778	****	162.78	****
PDI	0.857	0.832	30.478	****	60.21	***
ZP	0.645	0.583	15.272	****	18.94	****

Asterisks indicate the level of significance based on ANOVA, whereas **** *P* < 0.0001, *** 0.0001 ≤ *P* < 0.001, ** 0.001 ≤ *P* < 0.01, * 0.01 ≤ *P* < 0.05, and ns *P* ≥ 0.05. The difference between $\:{\mathrm{R}}_{\mathrm{a}\mathrm{d}\mathrm{j}}^{2}$ and $\:{\mathrm{R}}_{\mathrm{p}\mathrm{r}\mathrm{e}\mathrm{d}}^{2}$ must be <0.2

Evident differences were present between the three methods (Tables 4 and 5). In HSH, all factors significantly influenced PS, highlighting its high sensitivity, especially to the homogenization speed. In contrast, in DC method, PS was primarily driven by PVA concentration, with no influence from PLGA concentration and bead diameter. While in HPH, both PVA and PLGA concentrations were critical for PS values, with no influence from the pressure. On the other hand, PDI was consistently governed by PVA concentration in the three methods, confirming its role in system stabilization.

These findings provide further confirmation that the quality of nanoparticles obtained by the HSH method was more process-sensitive (in terms of particle size and homogeneity), as demonstrated by the highly significant impact of homogenization speed (Factor C) on PS in HSH (*P* ≤ 0.0001, Table 4), in contrast to DC and HPH where Factor C (bead diameter and pressure, respectively) showed no significant influence on PS (Table 4). This likely reflects the dominant role of shear-induced energy input in HSH emulsification, consistent with previous reports in the literature, emphasizing that in addition to the PVA and PLGA concentrations, the homogenization speed also has a significant influence on particle size [9, 14, 16]. In contrast, DC and HPH depended more on formulation parameters, particularly polymer-stabilizer interactions. The observed HPH behavior also aligns with the literature, where the formulation composition was more crucial for size control and stabilization rather than increasing the number of cycles [8, 9].

**Table 4:** Summary of the statistical significance of formulation and process factors on particle size (PS), polydispersity index (PDI), and zeta potential (ZP) in the high-shear homogenization (HSH), dual centrifugation (DC), and high-pressure homogenization (HPH) methods

Method	Factor A	Factor B	Factor C	
*HSH*	*PVA Concentration*	*PLGA Concentration*	*Homogenization Speed*	
PS	****	****	****	
PDI	****	****	ns	
ZP	****	ns	ns	
*DC*	*PVA Concentration*	*PLGA Concentration*	*Bead Diameter*	
PS	****	ns	ns	
PDI	****	ns	ns	
ZP	ns	***	**	
*HPH*	*PVA Concentration*	*PLGA Concentration*	*Pressure*	
PS	****	****	ns	
PDI	****	ns	ns	
ZP	ns	**	****	

Asterisks indicate the level of significance based on ANOVA, whereas **** *P* < 0.0001, *** 0.0001 ≤ *P* < 0.001, ** 0.001 ≤ *P* < 0.01, * 0.01 ≤ *P* < 0.05, and ns *P* ≥ 0.05

**Table 5:** The model equations in terms of coded factors capturing the effects of and interactions between formulation and process factors on particle size (PS), polydispersity index (PDI), and zeta potential (ZP) in the high-shear homogenization (HSH), dual centrifugation (DC), and high-pressure homogenization (HPH) methods

Method	Equation
*HSH*	
PS	137.96–15.74 A + 12.75B -10.82 C − 4.68AB − 6.88AC + 0.78BC + 26.86 A² + 2.51B² + 1.59 C²
PDI	0.05 + 0.01 A − 0.02B − 0.001 C − 0.01AB + 0.005AC − 0.003BC − 0.005A^2^ + 0.01B^2^ − 0.001C^2^
ZP	− 10.36 + 1.74 A − 0.43B − 0.33 C
*DC*	
PS	458.83–387.14 A − 22.44B − 78.22 C
PDI	0.16–0.22 A + 0.001B − 0.01 C − 0.08AB + 0.04AC + 0.03BC + 0.20A^2^ + 0.01B^2^ − 0.03C^2^
ZP	− 15.19–0.67 A − 1.52B − 1.15 C + 0.02AB + 1.90AC + 0.25BC + 0.31A^2^ − 1.78B^2^ + 1.38C^2^
*HPH*	
PS	207.66 + -62.59 A + 57.20B − 7.43 C − 29.49AB + 13.25AC − 6.34BC + 42.57A^2^ − 0.69B^2^ − 8.07C^2^
PDI	0.068–0.039 A + 0.04B − 0.01 C − 0.08AB + 0.03AC − 0.01BC + 0.04A^2^ + 0.03B^2^ − 6.71e-05C^2^
ZP	− 14.60 + 1.84 A + 1.24B + 3.01 C + 1.25AB − 1.13AC − 0.13BC − 1.63A^2^ − 1.22B^2^ + 1.43C^2^

##### Effect of factors on PLGA-NPs characteristics in HSH method

The RSM model for PS (Fig. 4; Table 5) demonstrated strong predictive performance, with a low coefficient of variation (3.36%), high agreement between $\:{\mathrm{R}}_{\mathrm{a}\mathrm{d}\mathrm{j}}^{2}$ (0.952) and $\:{\mathrm{R}}_{\mathrm{p}\mathrm{r}\mathrm{e}\mathrm{d}}^{2}$ (0.946) values with a difference of < 0.2, and a strong signal-to-noise ratio (adeq precision = 51). The model’s F-value of 195 confirmed that the model is highly significant, with an extremely low probability that such a large F-value occurred due to noise. However, the significant lack-of-fit indicated that additional influencing variables might exist beyond the modeled factors. The effects of formulation and process parameters on PS were highly significant, with PVA concentration (Factor A), PLGA concentration (Factor B), and homogenization speed (Factor C) all showing statistically significant influences (*P* < 0.0001). Specifically, higher PVA concentrations and faster homogenization speeds effectively reduced particle size, whereas increasing the PLGA concentration led to larger particles. Significant interactions between PVA and PLGA, as well as PVA and speed, further indicated that the size-reducing capacity of the stabilizer is highly dependent on both polymer load and mechanical shear.

The PDI model (Fig. 4; Table 5) exhibited moderate performance ($\:{\mathrm{R}}_{\mathrm{a}\mathrm{d}\mathrm{j}}^{2}$ = 0.651, $\:{\mathrm{R}}_{\mathrm{p}\mathrm{r}\mathrm{e}\mathrm{d}}^{2}$ = 0.597), with an adeq precision of 17 and a non-significant lack-of-fit, indicating that it effectively described the primary response trends. The model’s F-value (~19) confirmed the model’s strong significance and reliability. Both PVA and PLGA concentrations significantly influenced the PDI (*P* < 0.0001). Higher PLGA concentrations improved uniformity and reduced PDI, whereas excess PVA slightly increased it. However, homogenization speed had no significant effect (*P* = 0.4942) on PDI as a main factor, though significant interactive terms highlighted that optimal uniformity requires a delicate balance between stabilizer concentration and shear force. For ZP, the model (Fig. 4; Table 5) was statistically significant, but it exhibited lower explanatory power ($\:{\mathrm{R}}_{\mathrm{a}\mathrm{d}\mathrm{j}}^{2}$ = 0.417, $\:{\mathrm{R}}_{\mathrm{p}\mathrm{r}\mathrm{e}\mathrm{d}}^{2}$ = 0.372). The model’s F-value of ~22 confirmed the model’s significance with only a 0.01% chance that an F-value this large could occur due to noise. PVA concentration had the strongest effect on ZP (*P* < 0.0001), exhibiting a positive effect that shifted the surface charge toward less negative values, likely due to the shielding effect of the PVA layer on the nanoparticle surface. In contrast, PLGA concentration showed a marginally significant effect (*P* = 0.059). Homogenization speed was not a significant determinant of ZP (*P* = 0.139).

These findings highlighted the strong influence of formulation parameters, namely polymer and stabilizer concentrations (PLGA and PVA, respectively), on PS and PDI in the HSH method. Moreover, the homogenization speed was critical for reducing PS without affecting PDI or ZP. Convincingly, this analysis proved the superiority of the HSH method in preparing nanoparticles. Nonetheless, the findings also confirmed its reliance on homogenization speed, which may impact scalability and reproducibility across different settings.

##### Effect of factors on PLGA-NPs characteristics in DC method

For the DC method, the models (Fig. 4; Table 5) exhibited greater variability in their responses. The PS model demonstrated a low fit ($\:{\mathrm{R}}_{\mathrm{a}\mathrm{d}\mathrm{j}}^{2}$ = 0.259, $\:{\mathrm{R}}_{\mathrm{p}\mathrm{r}\mathrm{e}\mathrm{d}}^{2}$ = 0.233) with an adeq precision of ~9. The model’s F-value (~11) confirmed its statistical significance and robustness, given the unlikelihood that an F-value this large was due to noise. However, the high CV% (107.50%) hinted that additional process parameters may also be influencing the characteristics beyond the modeled factors. Among the formulation parameters studied, PVA concentration (Factor A) had a highly significant effect (*P* < 0.0001), demonstrating a strong negative correlation where a higher stabilizer concentration drastically reduced PS. In contrast, PLGA concentration (Factor B) and bead diameter (Factor C) were not significant (*P* = 0.741 and 0.247, respectively). This could be interpreted as that while the bead diameter might have influenced particle formation and size reduction, it was not the dominant determinant under the studied conditions.

The PDI model exhibited the strongest performance ($\:{\mathrm{R}}_{\mathrm{a}\mathrm{d}\mathrm{j}}^{2}$ = 0.608, $\:{\mathrm{R}}_{\mathrm{p}\mathrm{r}\mathrm{e}\mathrm{d}}^{2}$ = 0.567) with an adeq precision of ~16. The model’s F-value of ~33 confirmed the strong statistical significance. PVA concentration was again the most influential factor (*P* < 0.0001), sharply decreasing PDI to yield more monodisperse populations, whereas PLGA concentration (*P* = 0.946) and bead diameter (*P* = 0.707) were not significant. This further emphasized that stabilizer concentration was critical in controlling the polydispersity and stability of nanoparticle dispersion. In contrast, polymer concentration and processing parameters had a limited impact within the studied design space.

For ZP, the model exhibited lower explanatory power ($\:{\mathrm{R}}_{\mathrm{a}\mathrm{d}\mathrm{j}}^{2}$ = 0.280, $\:{\mathrm{R}}_{\mathrm{p}\mathrm{r}\mathrm{e}\mathrm{d}}^{2}$ = 0.204) yet retained a non-significant lack-of-fit, suggesting that despite the existing variability, the model remained adequate for assessing the influence of formulation parameters on ZP. The model’s F-value of ~5 confirmed its statistical significance. Unlike HSH, PVA concentration had no significant effect (*P* = 0.131), while PLGA concentration emerged as the most significant factor (*P* = 0.0008). The influence of bead diameter was also statistically significant (*P* = 0.0099). Both factors exhibited negative effects, resulting in more strongly negative surface charges at higher polymer levels and larger bead sizes. A highly significant interaction was also identified between PVA concentration and bead diameter, indicating that both polymer concentration and process conditions played a more critical role in governing surface charge in the DC method compared to the HSH method.

##### Effect of factors on PLGA-NPs characteristics in HPH method

In the HPH method, the RSM model for PS (Fig. 4; Table 5) showed a strong fit and explanatory power ($\:{\mathrm{R}}_{\mathrm{a}\mathrm{d}\mathrm{j}}^{2}$ = 0.942, $\:{\mathrm{R}}_{\mathrm{p}\mathrm{r}\mathrm{e}\mathrm{d}}^{2}$ = 0.931) with an adeq precision of ~47. The model’s F-value (~163) emphasized its statistical significance, indicating that there was only a 0.01% chance the result occurred due to noise. Similar to HSH, both PVA and PLGA concentrations had a strong influence on PS (*P* < 0.0001), with PVA significantly reducing size while PLGA increased it. In contrast, pressure by itself did not demonstrate statistical significance (*P* = 0.916). Highly significant interactive effects between all three parameters (PVA-PLGA, PVA-Pressure, and PLGA-Pressure) were observed. This outcome highlighted that in continuous HPH process, all parameters synergize and the applied shear force must be precisely matched to organic phase viscosity and stabilizer capacity. However, the model’s significant lack-of-fit hinted that while the model strongly explained the results, there could be other influences on the PS than the tested parameters.

In terms of PDI, the fit was strongly significant ($\:{\mathrm{R}}_{\mathrm{a}\mathrm{d}\mathrm{j}}^{2}$ = 0.857, $\:{\mathrm{R}}_{\mathrm{p}\mathrm{r}\mathrm{e}\mathrm{d}}^{2}$ = 0.832) with an adeq precision of ~30 and F-value of ~60. The PVA concentration was the only significant factor (*P* < 0.0001), exerting a positive effect where excess PVA increased polydispersity, while the PLGA concentration and pressure were not. Similar to PS, highly significant synergistic interactions among all variables were recorded for PDI. The lack-of-fit was also significant for this response. ZP showed a moderate fit with $\:{\mathrm{R}}_{\mathrm{a}\mathrm{d}\mathrm{j}}^{2}$ = 0.645 and $\:{\mathrm{R}}_{\mathrm{p}\mathrm{r}\mathrm{e}\mathrm{d}}^{2}$ = 0.583 and an adeq precision of ~15. The model’s F-value (~19) confirmed its significance relative to noise. PLGA concentration and pressure emerged as strong determinants of ZP (*P* = 0.0017 and <0.0001, respectively), both exerting significant positive effects that shifted ZP to be less negative. In contrast, PVA concentration did not have a statistically significant effect.

These findings collectively confirm the influence of stabilizer concentration (PVA) on obtaining homogeneous nanoparticles in terms of PS and PDI using the HPH method. The polymer concentration (PLGA) was critical for optimum PS and ZP. On the other hand, ZP was also influenced by the pressure used during homogenization. While ZP models showed lower predictive performance, ZP was retained as a contextual CQA to ensure physicochemical consistency of selected conditions rather than as a primary optimization driver.

#### Identification of optimal configurations

Based on statistical analyses and model validation outcomes, formulation selection was constrained within the predefined experimental levels to ensure robust and reliable identification of best performing combinations and avoid unreliable explorations beyond the experimental dataset. The predefined CQAs criteria were equally weighted and enforced obtaining PS between 100 and 200 nm, PDI between 0.01 and 0.1, and a strong negative ZP.

**Table 6:** Summary of the model outcomes showing the optimal values for PVA concentration (Factor A), PLGA concentration (Factor B), and the method-specific parameter (Factor C). Factor C was the homogenization speed (rpm) in high-shear homogenization (HSH), bead diameter (mm) in dual centrifugation (DC), and pressure (psi) in high-pressure homogenization (HPH)

Method	Factor A	Factor B	Factor C	Desirability
HSH	1% w/w	25.0 mg/mL	18,000 rpm	0.628
DC	5% w/w	14.5 mg/mL	0.8 mm	0.707
HPH	1% w/w	4.0 mg/mL	15,000 psi	0.711
	3% w/w	4.0 mg/mL	10,000 psi	0.706
	3% w/w	14.5 mg/mL	10,000 psi	0.656
	3% w/w	4.0 mg/mL	10,000 psi	0.632
	3% w/w	25.0 mg/mL	10,000 psi	0.611
	3% w/w	4.0 mg/mL	15,000 psi	0.604

The process resulted in selecting at least one favorable configuration from each preparation method, fulfilling the CQAs criteria. The optimal HSH condition was identified at the upper boundary of the studied design space (18,000 rpm), leaving limited operational headroom for scale transfer to larger instruments where achieving equivalent rotor tip speeds is technically constrained [14]. In contrast, the optimal conditions for DC and HPH were identified within mid-range or lower levels of their respective process parameters, indicating greater flexibility for scale transfer and process adjustment.

In the HSH method, one desirable formulation, whose levels were illustrated in Table 6, was identified. The PVA concentration was at the lowest level, while the PLGA concentration and homogenization speed were at their highest levels. This outcome further emphasized the critical importance of homogenization speed for the quality of nanoparticles prepared by HSH. In the case of DC, the method showed less reliance on process parameters and more on formulation parameters in the selected formulation. The bead diameter was at its lowest level, while the PVA concentration was at its highest level, and the PLGA concentration was at an intermediate level. For the nanoparticles prepared by HPH, the optimization algorithm selected six formulations with desirability ranging from 0.604 to 0.711. In all six of them, the PVA concentration was either at the lowest or middle level, while the PLGA concentration varied across all its levels. Moreover, the pressure values were also at their lowest and middle levels, suggesting less sensitivity to process parameters and a greater reliance on formulation parameters. Notably, no confirmatory experiments were conducted at the identified conditions, as validation of predictive optimality was beyond the scope of this comparative study.

## Discussion

The development of PLGA-NPs has been a strong enabler for personalized and precision medicine with enhanced control over drug delivery [3, 4, 20]. However, the success of these systems depends not only on their physicochemical properties but also on the robustness, reproducibility, and regulatory compliance of their preparation processes. While HSH has historically been a widely established standard for PLGA-NPs production, its inherent limitations, including high sensitivity to process-specific parameters (principally homogenization speed) that introduces scale-dependent variability during technology transfer, difficulty in complying with continuous manufacturing requirements, reliance on high-energy emulsification, and potential contamination risks, necessitate exploring alternative and more adaptable methodologies [14].

This study introduces DC as a novel alternative preparation method for PLGA-NPs, employing a statistical approach to systematically optimize process parameters. To the best of our knowledge, this is the first report demonstrating DC as an efficient and reliable approach to PLGA-NPs preparation, aligning with the regulatory guidelines on pharmaceutical nanotechnology [33]. DC can employ sterile, in-vial homogenization, where the formulation is processed within a sealed, contamination-free system, mitigating risks associated with environmental exposure and cross-contamination [23]. Moreover, it has a distinct advantage over other homogenization methods since it requires no dead volume for homogenization, allowing very small batches to be processed unlike HSH and HPH [23]. Notably, DC delivers a much lower peak energy distributed over thousands of consecutive homogenization events through beads movements and collisions, resulting in overall gentler but cumulatively effective processing [23]. This minimal mechanical stress and controlled energy input can enable optimized drug encapsulation for fragile and labile therapeutics, which makes DC particularly attractive for personalized medicine, where patient-specific formulations are required, and the conventional methods fall short. However, the low model fit observed in DC could potentially hint at the inherently stochastic nature of bead-mediated size reduction, where particle formation depends not only on the studied factors but also on bead-bead and bead-vial collision dynamics and local energy dissipation [23]. These intricate interactions are difficult to capture within a second-order polynomial framework. Higher-order interactions or additional process parameters would likely be necessary to substantially improve model predictability and represent a priority for future work.

The study also validated HPH as a viable method for producing PLGA-NPs. HPH combines the benefits of consistency, closed-system handling, and compatibility with continuous manufacturing [14, 23]. HPH employs intense pressure to achieve size reduction in a more controlled and continuous manner [10, 19, 22, 37, 38]. In contrast to HSH and DC, HPH applies very high peak energy for millisecond-scale homogenization cycles that only come in contact with samples for a limited time [23]. Moreover, HPH can be operated in continuous manner, making it readily compliant with regulatory mandates for continuous manufacturing [14, 18, 19]. In this study, HPH yielded NPs with good characteristics and CQAs, closer to HSH in terms of median values but with a wider breadth. Importantly, both DC and HPH offer cleaner and modular processing environments. This advantage positions them as competent candidates for modern pharmaceutical workflows that prioritize automation, in-line control, digitalization, and machine learning, as well as flexible production scales, in line with recent ICH guidelines [19, 39–41].

The optimization approach highlighted that HSH was process-sensitive and highly influenced by the source and amount of applied shear energy through homogenization [9, 14]. In contrast, both DC and HPH methods were formulation-composition-sensitive, where the balance between polymer and stabilizer concentrations played a critical role in the quality of the prepared PLGA-NPs. These findings align with the literature on HPH, where increasing the number of homogenization cycles did not enhance PS or PDI [8, 9]. Overall, in all methods, PVA concentration was the most significant factor that strongly influenced formulation’s characteristics. This observation closely aligns with previous studies that demonstrate the strong influence of stabilizer concentration on nanoparticle properties and surface charge [3, 42].

With regards to potential applications in precision medicine, the defined QTPP thresholds reflect well-established functional requirements for PLGA-NPs as drug delivery systems. PS is known to directly influence distribution and cellular uptake. Nanoparticles < 200 nm are generally associated with favorable passive targeting through the enhanced permeability and retention (EPR) effect, while those > 250 nm are more susceptible to rapid mononuclear phagocyte system clearance [2, 8]. A shift in median PS from 150 to 200 nm, as observed between HSH and HPH, remains within the therapeutically acceptable window and does not constitute a functional disadvantage. Similarly, PDI below 0.3 ensures homogeneity and predictable in vivo behavior [33]. The consistently negative ZP across all methods most likely reflects the deprotonation of PLGA’s terminal carboxylic groups and contributes to colloidal stability through electrostatic repulsion [34]. Although ZP’s value varied across experimental conditions, all methods produced formulations with sufficient surface charge to prevent immediate aggregation and ensure stability.

Collectively, this study underscores the need for deeper, application-specific optimization of both formulation and process parameters in PLGA-NPs preparation to tailor the resulting PLGA-NPs quality for the intended application or scale. Specifically, when it comes to DC. While the results proved that DC is a capable and competent small-batch method for PLGA-NPs preparation, they also shed light on the higher variability in characterization results, signaling a substantial room for improvement. Therefore, this work opens the door for further investigation and refinement in formulation composition (polymer-stabilizer ratio) and process parameters, including centrifugation time, effective centrifugal force, bead loading, and different diameters. Furthermore, leveraging advances in computational modeling, molecular dynamics simulations, and machine learning could enhance the mechanistic understanding of how other CPPs in the formulation and preparation method influence the CQAs of PLGA-NP, providing more guidance for developing next-generation therapeutics [43].

## Conclusion

This study established DC and HPH as viable alternatives to HSH for the preparation of PLGA-based nanobiomaterials. Using a systematic approach, we were able to identify the optimal levels for key formulation and process parameters to ensure reproducibility. While HSH offered strong control over particle attributes, the inherent limitations of high-shear probes reduce its scalability potential. DC exhibited higher variability but can be readily optimized for small-batch, patient-specific production. In contrast, HPH delivered consistent outcomes, with superior scalability for continuous manufacturing applications. These findings highlight the potential of DC and HPH to support regulatory-compliant nanomedicine manufacturing. Beyond addressing manufacturing challenges, the established framework lays the foundation for further expansion into the development of multifunctional, stimuli-responsive, and computationally-optimized nanoparticles tailored for nanomedical applications. The platform’s adaptability also enables its application to diverse payloads and polymer systems, bridging formulation innovation with clinical and industrial deployment.

## Supplementary Information

Below is the link to the electronic supplementary material.

Supplementary Material 1

## Data Availability

Data will be made available on request from the authors.
