# Supplementary material for: Systematic evaluation and statistical modeling of dual centrifugation and high-pressure homogenization as promising methods for scalable preparation of polymeric nanoparticles
Source: Discov Nano. 2026 Jul 17;21(1):348. doi: 10.1186/s11671-026-04808-y (PMC13376087; doi:10.1186/s11671-026-04808-y)
Supplement: Supplementary file 1 — Supplementary Material 1 [file 11671_2026_4808_MOESM1_ESM.docx]

**Supplementary Information**

Systematic Evaluation and Statistical Modeling of Dual Centrifugation and High-Pressure Homogenization as Promising Methods for Scalable Preparation of Polymeric Nanoparticles

**Amr Ehab Kamel^1,2^, Stefanie Klein^1^, Lisa Schöne^1^, Sara Gabr^1,3^, Udo Bakowsky^1^, Jens Schäfer^1*^**

^1^ Philipps-Universität Marburg, Department of Pharmacy, Institute for Pharmaceutics and Biopharmaceutics, Robert-Koch-Str. 4, 35037 Marburg, Germany; ^2^ Department of Pharmaceutics and Pharmaceutical Technology, Faculty of Pharmacy, Heliopolis University, Cairo, Egypt; ^3^ Department of Pharmacognosy, Faculty of Pharmacy, Heliopolis University, Cairo, Egypt

# 1. Physicochemical Characterization

Table S1. Physicochemical characterization results for the 90 randomized runs in the experimental dataset. Factors: PVA concentration (Factor A; % w/w), PLGA concentration (Factor B; mg/mL), and the coded method-specific parameter (Factor C). Factor C in HSH was the homogenization speed (rpm), where -1 (12,000), 0 (15,000), and 1 (18,000) were the levels. In DC, it was bead diameter (mm), where -1 (0.8), 0 (1.1), and 1 (1.5) were the levels. Finally, in HPH, it was pressure (psi) in HPH, where -1 (10,000), 0 (15,000), and 1 (20,000) were the levels.

| **Run** | **Factor** | | | **PS** | | | **PDI** | | | **ZP** | | |
| --- | --- | --- | --- | --- | --- | --- | --- | --- | --- | --- | --- | --- |
|  | A | B | C | HSH | DC | HPH | HSH | DC | HPH | HSH | DC | HPH |
| 31 | 1 | 4 | -1 | 166.80 | 2143.33 | 210.97 | 0.074 | 0.756 | 0.060 | -11.24 | -10.87 | -23.40 |
| 61 | 1 | 4 | -1 | 165.93 | 1918.33 | 213.53 | 0.038 | 0.656 | 0.056 | -9.50 | -10.60 | -16.90 |
| 1 | 1 | 4 | -1 | 160.57 | 1576.67 | 210.90 | 0.043 | 0.730 | 0.041 | -11.77 | -11.10 | -19.23 |
| 70 | 1 | 4 | 0 | 158.57 | 295.93 | 229.30 | 0.068 | 0.205 | 0.031 | -12.03 | -15.13 | -18.63 |
| 40 | 1 | 4 | 0 | 158.67 | 291.53 | 224.17 | 0.067 | 0.196 | 0.060 | -10.87 | -16.43 | -19.13 |
| 10 | 1 | 4 | 0 | 154.93 | 2424.67 | 212.13 | 0.037 | 0.999 | 0.047 | -14.12 | -14.40 | -19.00 |
| 49 | 1 | 4 | 1 | 160.30 | 262.23 | 214.33 | 0.029 | 0.166 | 0.044 | -12.37 | -15.10 | -14.83 |
| 19 | 1 | 4 | 1 | 152.00 | 305.03 | 219.57 | 0.040 | 0.213 | 0.043 | -17.27 | -15.70 | -13.77 |
| 79 | 1 | 4 | 1 | 153.83 | 582.07 | 218.77 | 0.073 | 0.407 | 0.042 | -14.33 | -14.17 | -15.87 |
| 64 | 1 | 14.5 | -1 | 186.20 | 422.90 | 299.80 | 0.043 | 0.395 | 0.183 | -10.66 | -13.47 | -19.73 |
| 4 | 1 | 14.5 | -1 | 183.27 | 714.40 | 330.47 | 0.058 | 0.345 | 0.194 | -11.33 | -0.15 | -19.46 |
| 34 | 1 | 14.5 | -1 | 182.20 | 2141.67 | 307.67 | 0.039 | 1.000 | 0.174 | -11.37 | 0.15 | -19.83 |
| 13 | 1 | 14.5 | 0 | 181.60 | 497.30 | 315.30 | 0.033 | 0.443 | 0.187 | -12.90 | -16.67 | -18.87 |
| 43 | 1 | 14.5 | 0 | 181.47 | 1165.67 | 335.87 | 0.017 | 0.661 | 0.214 | -12.00 | -14.83 | -21.53 |
| 73 | 1 | 14.5 | 0 | 183.70 | 813.63 | 322.30 | 0.017 | 0.643 | 0.193 | -11.77 | -16.17 | -21.40 |
| 52 | 1 | 14.5 | 1 | 173.30 | 1094.03 | 262.10 | 0.013 | 0.674 | 0.103 | -11.97 | -15.57 | -11.43 |
| 22 | 1 | 14.5 | 1 | 182.40 | 1429.67 | 274.30 | 0.020 | 0.696 | 0.086 | -12.10 | -14.93 | -10.80 |
| 82 | 1 | 14.5 | 1 | 181.17 | 529.20 | 272.73 | 0.016 | 0.452 | 0.132 | -11.30 | -17.20 | -14.83 |
| 37 | 1 | 25 | -1 | 204.40 | 835.30 | 436.97 | 0.042 | 0.682 | 0.368 | -13.20 | -14.77 | -22.40 |
| 7 | 1 | 25 | -1 | 195.23 | 843.43 | 410.87 | 0.037 | 0.678 | 0.291 | -12.93 | -15.33 | -20.63 |
| 67 | 1 | 25 | -1 | 205.37 | 704.77 | 476.93 | 0.032 | 0.565 | 0.458 | -13.37 | -14.73 | -24.23 |
| 76 | 1 | 25 | 0 | 201.63 | 703.70 | 404.10 | 0.026 | 0.559 | 0.301 | -13.00 | -18.20 | -22.23 |
| 16 | 1 | 25 | 0 | 198.00 | 723.20 | 405.53 | 0.058 | 0.631 | 0.277 | -11.10 | -18.63 | -22.47 |
| 46 | 1 | 25 | 0 | 201.53 | 762.53 | 431.27 | 0.020 | 0.623 | 0.361 | -11.04 | -17.83 | -21.47 |
| 85 | 1 | 25 | 1 | 194.93 | 698.13 | 339.03 | 0.041 | 0.626 | 0.214 | -12.70 | -16.87 | -11.03 |
| 25 | 1 | 25 | 1 | 193.03 | 1103.67 | 327.37 | 0.021 | 0.788 | 0.220 | -12.80 | -19.47 | -10.25 |
| 55 | 1 | 25 | 1 | 197.63 | 712.13 | 381.97 | 0.036 | 0.580 | 0.243 | -12.57 | -18.43 | -10.60 |
| 2 | 3 | 4 | -1 | 147.47 | 139.80 | 156.03 | 0.058 | 0.054 | 0.059 | -9.58 | -15.07 | -25.10 |
| 32 | 3 | 4 | -1 | 140.23 | 158.47 | 159.47 | 0.072 | 0.105 | 0.082 | -7.62 | -12.43 | -19.47 |
| 62 | 3 | 4 | -1 | 147.10 | 157.47 | 153.80 | 0.075 | 0.132 | 0.045 | -9.14 | -12.70 | -15.70 |
| 41 | 3 | 4 | 0 | 128.67 | 199.30 | 164.10 | 0.086 | 0.248 | 0.078 | -8.72 | -13.90 | -13.80 |
| 11 | 3 | 4 | 0 | 128.13 | 148.93 | 156.83 | 0.067 | 0.183 | 0.083 | -10.79 | -15.13 | -16.00 |
| 71 | 3 | 4 | 0 | 121.23 | 150.40 | 148.43 | 0.057 | 0.167 | 0.047 | -9.11 | -13.80 | -14.17 |
| 20 | 3 | 4 | 1 | 117.93 | 156.73 | 155.53 | 0.072 | 0.134 | 0.101 | -9.95 | -16.23 | -15.00 |
| 80 | 3 | 4 | 1 | 122.83 | 146.70 | 154.97 | 0.092 | 0.131 | 0.083 | -5.42 | -15.27 | -14.00 |
| 50 | 3 | 4 | 1 | 114.07 | 171.77 | 143.70 | 0.053 | 0.201 | 0.078 | -6.66 | -15.23 | -12.67 |
| 65 | 3 | 14.5 | -1 | 146.07 | 199.93 | 209.63 | 0.043 | 0.033 | 0.076 | -9.41 | -14.57 | -14.93 |
| 35 | 3 | 14.5 | -1 | 159.43 | 196.80 | 211.20 | 0.025 | 0.044 | 0.079 | -10.69 | -13.13 | -15.80 |
| 5 | 3 | 14.5 | -1 | 145.17 | 197.70 | 211.47 | 0.057 | 0.055 | 0.064 | -10.18 | -13.67 | -16.30 |
| 60 | 3 | 14.5 | 0 | 139.03 | 265.80 | 214.33 | 0.053 | 0.184 | 0.069 | -9.37 | -16.10 | -13.77 |
| 58 | 3 | 14.5 | 0 | 151.67 | 198.57 | 212.40 | 0.053 | 0.066 | 0.091 | -10.12 | -16.93 | -16.97 |
| 14 | 3 | 14.5 | 0 | 126.07 | 208.57 | 210.03 | 0.062 | 0.121 | 0.066 | -13.60 | -15.90 | -15.60 |
| 30 | 3 | 14.5 | 0 | 138.93 | 277.63 | 208.43 | 0.054 | 0.141 | 0.071 | -9.80 | -15.90 | -15.70 |
| 59 | 3 | 14.5 | 0 | 137.50 | 3510.33 | 210.53 | 0.068 | 1.000 | 0.072 | -8.91 | 0.04 | -15.97 |
| 28 | 3 | 14.5 | 0 | 143.03 | 202.73 | 205.27 | 0.047 | 0.056 | 0.023 | -8.99 | -16.03 | -15.13 |
| 90 | 3 | 14.5 | 0 | 139.93 | 261.13 | 210.73 | 0.030 | 0.132 | 0.070 | -9.41 | -15.03 | -16.40 |
| 29 | 3 | 14.5 | 0 | 132.80 | 201.03 | 195.73 | 0.042 | 0.038 | 0.049 | -11.28 | -16.53 | -15.87 |
| 89 | 3 | 14.5 | 0 | 129.80 | 234.87 | 197.87 | 0.050 | 0.119 | 0.061 | -9.36 | -15.97 | -15.57 |
| 74 | 3 | 14.5 | 0 | 128.60 | 213.20 | 211.03 | 0.048 | 0.069 | 0.076 | -12.07 | -15.57 | -15.80 |
| 44 | 3 | 14.5 | 0 | 136.37 | 261.40 | 198.50 | 0.048 | 0.159 | 0.068 | -12.97 | -16.43 | -16.20 |
| 88 | 3 | 14.5 | 0 | 138.67 | 231.77 | 190.00 | 0.042 | 0.086 | 0.069 | -11.53 | -16.23 | -10.63 |
| 83 | 3 | 14.5 | 1 | 125.83 | 195.33 | 199.43 | 0.052 | 0.062 | 0.083 | -9.23 | -17.23 | -10.11 |
| 53 | 3 | 14.5 | 1 | 129.60 | 217.40 | 204.40 | 0.056 | 0.092 | 0.054 | -10.29 | -16.40 | -9.84 |
| 23 | 3 | 14.5 | 1 | 129.20 | 197.43 | 201.40 | 0.040 | 0.065 | 0.063 | -12.03 | -17.40 | -10.60 |
| 8 | 3 | 25 | -1 | 150.67 | 244.60 | 230.43 | 0.045 | 0.120 | 0.102 | -10.43 | -16.13 | -16.43 |
| 68 | 3 | 25 | -1 | 165.83 | 265.00 | 257.30 | 0.028 | 0.160 | 0.147 | -10.07 | -15.87 | -15.80 |
| 38 | 3 | 25 | -1 | 152.83 | 263.63 | 293.40 | 0.048 | 0.156 | 0.190 | -10.68 | -15.23 | -16.63 |
| 17 | 3 | 25 | 0 | 143.93 | 270.83 | 217.20 | 0.040 | 0.142 | 0.069 | -12.27 | -18.20 | -15.17 |
| 77 | 3 | 25 | 0 | 142.80 | 297.37 | 259.10 | 0.051 | 0.210 | 0.146 | -9.65 | -18.27 | -16.50 |
| 47 | 3 | 25 | 0 | 147.50 | 301.00 | 244.30 | 0.041 | 0.228 | 0.117 | -10.25 | -16.70 | -8.54 |
| 56 | 3 | 25 | 1 | 133.30 | 248.27 | 232.13 | 0.035 | 0.098 | 0.119 | -10.56 | -18.27 | -8.73 |
| 86 | 3 | 25 | 1 | 133.40 | 295.70 | 243.43 | 0.041 | 0.154 | 0.143 | -12.57 | -16.10 | -8.94 |
| 26 | 3 | 25 | 1 | 134.40 | 324.70 | 245.37 | 0.022 | 0.215 | 0.155 | -10.45 | -16.87 | -7.92 |
| 3 | 5 | 4 | -1 | 150.40 | 123.13 | 126.00 | 0.080 | 0.133 | 0.080 | -7.85 | -13.00 | -20.87 |
| 63 | 5 | 4 | -1 | 155.27 | 157.65 | 162.20 | 0.077 | 0.278 | 0.138 | -11.26 | -13.45 | -23.27 |
| 33 | 5 | 4 | -1 | 157.53 | 118.93 | 135.23 | 0.067 | 0.072 | 0.117 | -5.64 | -15.27 | -23.33 |
| 12 | 5 | 4 | 0 | 134.37 | 133.43 | 168.73 | 0.074 | 0.242 | 0.158 | -8.14 | -15.13 | -16.03 |
| 72 | 5 | 4 | 0 | 133.30 | 210.90 | 160.70 | 0.089 | 0.465 | 0.150 | -8.77 | -13.07 | -17.10 |
| 42 | 5 | 4 | 0 | 137.13 | 130.35 | 138.57 | 0.076 | 0.197 | 0.096 | -8.04 | -16.17 | -14.40 |
| 81 | 5 | 4 | 1 | 117.57 | 126.93 | 139.77 | 0.093 | 0.130 | 0.139 | -6.42 | -13.83 | -14.70 |
| 51 | 5 | 4 | 1 | 120.23 | 241.70 | 140.37 | 0.105 | 0.367 | 0.135 | -15.29 | -14.23 | -15.07 |
| 21 | 5 | 4 | 1 | 119.83 | 126.50 | 146.37 | 0.096 | 0.161 | 0.165 | -7.19 | -17.07 | -12.73 |
| 6 | 5 | 14.5 | -1 | 168.40 | 157.87 | 180.20 | 0.053 | 0.067 | 0.057 | -10.66 | -17.27 | -12.83 |
| 36 | 5 | 14.5 | -1 | 168.80 | 185.95 | 194.50 | 0.048 | 0.188 | 0.077 | -8.93 | -18.08 | -13.47 |
| 66 | 5 | 14.5 | -1 | 170.23 | 163.27 | 188.27 | 0.055 | 0.033 | 0.067 | -7.80 | -16.63 | -14.90 |
| 45 | 5 | 14.5 | 0 | 151.87 | 171.20 | 188.43 | 0.048 | 0.100 | 0.056 | -9.09 | -15.40 | -11.50 |
| 75 | 5 | 14.5 | 0 | 150.73 | 155.03 | 196.87 | 0.064 | 0.030 | 0.065 | -8.38 | -16.07 | -14.50 |
| 15 | 5 | 14.5 | 0 | 154.40 | 168.73 | 190.40 | 0.051 | 0.086 | 0.053 | -8.39 | -16.90 | -13.33 |
| 84 | 5 | 14.5 | 1 | 134.83 | 172.60 | 174.30 | 0.035 | 0.090 | 0.046 | -7.06 | -16.53 | -10.19 |
| 54 | 5 | 14.5 | 1 | 134.07 | 159.27 | 185.27 | 0.057 | 0.048 | 0.076 | -8.02 | -17.90 | -9.68 |
| 24 | 5 | 14.5 | 1 | 132.40 | 297.23 | 175.47 | 0.060 | 0.351 | 0.061 | -8.49 | -0.07 | -9.72 |
| 9 | 5 | 25 | -1 | 171.67 | 197.13 | 207.47 | 0.027 | 0.021 | 0.067 | -7.77 | -17.70 | -12.43 |
| 69 | 5 | 25 | -1 | 175.33 | 203.43 | 185.90 | 0.053 | 0.041 | 0.059 | -9.34 | -17.70 | -12.20 |
| 39 | 5 | 25 | -1 | 176.00 | 204.47 | 182.63 | 0.044 | 0.060 | 0.053 | -6.47 | -19.27 | -12.73 |
| 18 | 5 | 25 | 0 | 155.07 | 200.97 | 218.10 | 0.036 | 0.045 | 0.074 | -9.76 | -17.50 | -11.93 |
| 48 | 5 | 25 | 0 | 155.83 | 193.73 | 220.57 | 0.042 | 0.078 | 0.103 | -9.66 | -17.60 | -12.37 |
| 78 | 5 | 25 | 0 | 157.50 | 205.43 | 212.27 | 0.027 | 0.051 | 0.050 | -9.64 | -17.50 | -13.17 |
| 27 | 5 | 25 | 1 | 139.43 | 193.57 | 238.73 | 0.041 | 0.086 | 0.127 | -8.96 | -17.17 | -13.60 |
| 57 | 5 | 25 | 1 | 142.13 | 185.77 | 223.80 | 0.039 | 0.044 | 0.110 | -9.96 | -15.83 | -14.13 |
| 87 | 5 | 25 | 1 | 144.27 | 204.60 | 227.23 | 0.025 | 0.080 | 0.115 | -10.90 | -21.13 | -14.47 |





Fig. S1. Histograms showing the frequency distribution of physicochemical characterization results (PS, PDI, and ZP) in the three methods.

# 2. Principal Component Analysis (PCA)

Principal Component Analysis was performed to explore trends, patterns, and correlations across the whole dataset of PS, PDI, and ZP measurements pooled from all experimental runs for HSH, DC, and HPH methods. Data were standardized to have a mean of 0 (zero) and a standard deviation of 1 (one), and variables were equally weighted prior to analysis to account for scale differences. PCA was conducted on the correlation matrix, and the Principal Components (PCs) were determined by parallel analysis. The resulting PCs and their associated results were interpreted to identify the primary sources of variability and to facilitate direct comparison and clustering of formulations across preparation methods. The findings of PCA provide a complementary level of insight beyond factorial modeling by revealing the latent structures within the dataset and highlighting relationships that might not be directly observable from raw response values.

PCA is a powerful unsupervised multivariate analysis method widely recognized for its ability to reduce and interpret complex, multidimensional data generated during the design and evaluation of complex systems, such as pharmaceutical formulations. By reducing multiple formulation and process parameters into a small set of Principal Components (PCs), PCA can efficiently detect the trends in data and provide meaningful outcomes on the dominant influences as well as sources of variability with improved data visualization. This dimensionality reduction streamlines analysis and helps uncover hidden trends, which supports decision-making throughout formulation development. PCA was conducted on the combined datasets for each of the CQAs (PS, PDI, and ZP) obtained from the 90 experimental runs across the three preparation methods (HSH, DC, and HPH).

For PS, the eigen decomposition of the standardized covariance matrix yielded three Principal Components, which cumulatively accounted for 100% of the total variance in the dataset. Parallel analysis was employed to objectively determine the number of significant components to retain (i.e., the threshold). The first Principal Component (PC1) showed a significant eigenvalue of 2.042, which exceeded the threshold (1.166 – 1.315). This confirmed the relevance of PC1 in capturing the underlying variance in the dataset (Fig. 2). In contrast, the second (PC2) and third (PC3) Principal Components exhibited eigenvalues below their respective benchmarks and were therefore excluded from further analysis. Notably, PC1 accounted for 68.08% of the total variance, while PC2 and PC3 collectively explained the remaining 31.92%.

Analyzing the loading matrix revealed that HSH (0.916) and HPH (0.907) exhibited the strongest associations with PC1, indicating their significant influence on PS variability. In comparison, DC contributed less to PC1, with a loading of 0.618. The relative contributions of each method to PC1 further highlighted the dominant influences of HSH (41.0%) and HPH (40.3%), while DC accounted for a smaller contribution of 18.7%. The eigenvector coefficients for PC1 were 0.641 (HSH), 0.432 (DC), and 0.635 (HPH), reflecting comparable contributions for HSH and HPH in defining PC1. These outcomes reinforced the earlier findings in the RSM analysis that highlighted the similarities between these two methods in their effects on PS.

For PDI, the eigen decomposition of the standardized covariance matrix also yielded three PCs. Parallel analysis determined that PC1 was the only significant component (Fig. 2), with an eigenvalue of 1.643 (Threshold: 1.064–1.295). The loadings on PC1 showed that HSH contributed positively (0.565), whereas both DC (-0.807) and HPH (-0.820) contributed negatively, indicating their inverse influence on PDI. The contributions of variables to PC1 indicated that HSH accounted for 19.4%, DC for 39.6%, and HPH for 40.9%. This highlighted that the primary sources of variability in PDI are associated with DC and HPH, while HSH showed more consistency and less variability. For ZP, PCA did not yield any significant PCs based on parallel analysis, as none of the eigenvalues exceeded the threshold. This indicated that ZP values across the three methods were uncorrelated and lacked a common underlying variance structure. The absence of common trends suggested that ZP properties could be method-specific and not influenced by a dominant latent factor.

Overall, PCA offered complementary insights into DoE and RSM by identifying the method-specific variance trends. PS was primarily influenced by HSH and HPH, indicating that these methods exhibited consistent performance. In contrast, PDI variability was negatively influenced by DC and HPH, confirming the previous findings in RSM analysis and highlighting that there is still room for optimization in both methods. Finally, ZP showed no clear trend in variability, suggesting independent behavior across methods. Although PCA by itself does not pinpoint optimal formulation or process parameters, the outcomes provided a meaningful contribution to demystify some of the observations in RSM analysis and reduce the unexplained variability.


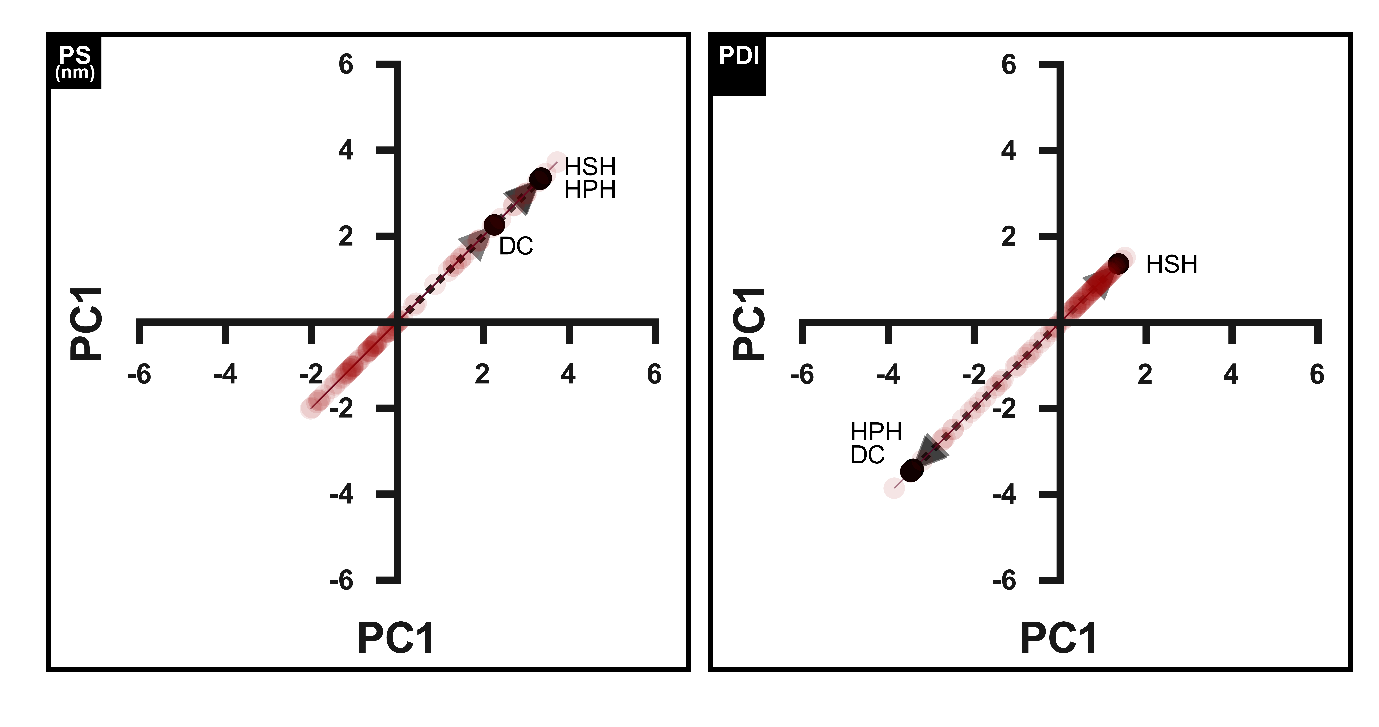


Fig. S2 | Principal Component Analysis (PCA) biplot showing PC1 scores (red, transparent circles and lines) and variable loadings (black, dotted line with arrowheads) for particle size (PS) and polydispersity index (PDI) across the three preparation methods.
